# Maladie de Still de l’adulte et lymphome: une association rare

**DOI:** 10.11604/pamj.2020.36.55.9806

**Published:** 2020-06-02

**Authors:** Jihane Smaali, Abdessadak El Khattabi, Mohamed El Qatni, Fadoua Mekouar, Youssef Sekkach, Ali Abouzahir, Taoufik Ameziane, Driss Ghafir

**Affiliations:** 1 Service de Médecine Interne B, Hôpital Militaire d’Instruction Mohamed V, Rabat, Maroc

**Keywords:** Maladie de Still de l’adulte, lymphome, cancer, maladies auto-immunes, hémopathies, Adult onset Still′s disease, lymphoma, cancer, autoimmune diseases, hematopathies

## Abstract

La maladie de Still de l’adulte (MSA) et les lymphomes sont des pathologies dont la présentation clinique et même histopathologique est très proche. L′association des deux pathologies est rarement rapportée dans la littérature. Nous rapportons une observation de maladie de Still de l′adulte diagnostiquée chez une patiente de 26 ans antérieurement traitée pour lymphome malin non hodgkinien (LMNH) à grandes cellules B par chimiothérapie et auto-greffe de cellules souches hématopoïétiques avec rémission complète. Les cas de MSA associés aux lymphomes sont rares et dans tous les cas le diagnostic de MSA avait précédé celui de lymphome. Notre observation est particulière par la succession LMNH puis MSA et soulève plusieurs hypothèses sur les liens entre ces deux pathologies. Les liens entre maladies dysimmunitaires et hémopathies lymphoïdes ont largement été prouvés, qu′il s′agisse de l′évolution de maladies auto-immunes vers un lymphome ou de manifestations dysimmunitaires survenant au cours de ce dernier. Notre cas illustre la difficulté de distinction entre ces entités.

## Abstract (en)

Adult onset Still′s disease (AOSD) and lymphomas are diseases characterized by very similar clinical and histopathological manifestations. The association between these diseases has rarely been reported in the literature. We here report the case of a 26-year old patient diagnosed with adult onset Still′s disease. She had been previously treated for large B-cell Non-Hodgkin lymphoma (LBCNHL) with chemotherapy and haemopoietic stem-cell autograft with complete remission. The association between AOSD and lymphoma is rare and in all cases AOSD has been diagnosed before lymphoma. The peculiarity of this study lies in the succession LBCNHL- AOSD and raises several hypotheses on the association between these two diseases. The association between non-autoimmune diseases and lymphoid hemopathies have been widely demonstrated, whether it is the progression of autoimmune diseases vs lymphoma or non-autoimmune disease manifestations occurring in patients with this disease. This study highlights the difficult distinction between these diseases.

## Introduction

La maladie de Still de l’adulte est une maladie inflammatoire rare qui touche le plus souvent des sujets âgés de 16 à 35 ans. Ses manifestations cliniques sont très polymorphes et le diagnostic est retenu sur un faisceau d’arguments clinico-biologiques et l’exclusion d’une autre pathologie infectieuse, immunologique ou néoplasique. L’association maladie de Still et lymphome est rarement rapportée dans la littérature surtout que la distinction entre ces deux entités est difficile. Nous rapportons une observation particulière de maladie de Still de l’adulte diagnostiquée chez une patiente déjà traitée pour LMNH.

## Patient et observation

Patiente âgée de 26 ans sans antécédents pathologiques notables et qui a présenté en juillet 2011 une polyarthrite fébrile avec éruption maculo-papuleuse évanescente au niveau des coudes, des bras et du cou ainsi qu’une polyadénopathie cervicale évoluant dans un contexte d’altération de l’état général.

Le bilan initial objectivait un syndrome inflammatoire (VS: 47mm, CRP: 28 mg/l, Fg: 8,21g/l, EPP: hypoalbuminémie à 26,1g/l avec hyperalpha1, béta1 et hypergammaglobulinémie polyclonale, l2 hémogramme était normal avec Hb: 13,2 g/dl, GB: 9600 élts/mm^3^, PNN: 5600 élts/mm^3^, Lymphocytes: 1610 élts/mm^3^, PLq: N, Ferritinémie: 325 ng/ml, LDH: 500 UI/l, le bilan infectieux et les sérologies des hépatites B et C, EBV, CMV et VIH étaient négatives de même que le bilan immunologique (Ac antinucléaires, ANCA et anti-CCP). La biopsie ganglionnaire avait révélé un lymphome diffus à grandes cellules B (CD20+,CD5 faible et cycline D1-) avec au bilan d’extension (TDM étagée: adénopathies sus et sous diaphragmatiques, FOGD: antrite nodulaire avec à la biopsie un aspect inflammatoire et à la BOM on ne notait pas d’envahissement). Le diagnostic de lymphome diffus à grandes cellules B stade IIIBb, aaIPI = 2 a été posé.

La patiente a alors été mise sous protocole R-CHOP avec réponse partielle puis sous protocole R-ECHAP avec obtention d’une rémission complète consolidée par une auto-greffe de cellules souches en Mars 2012. Elle a présenté 18 mois plus tard une polyarthrite fébrile avec éruption maculopapuleuse évanescente au niveau des bras et du cou et des adénopathies latérocervicales bilatérales. Au bilan on notait un syndrome inflammatoire important (VS: 90mm, CRP: 76 mg/l, Fg: 6g/l, EPP: profil inflammatoire avec hypoalbuminémie, à l’hémogramme on notait une hyperleucocytose à 13800 élts/mm^3^ à prédominance PNN: 10900 élts/mm^3^, anémie avec Hb: 11,2g/dl normocytaire PLQ: N, Ferritinémie > 1500ng/ml, LDH: 200 UI/l, le bilan rénal et hépatique étaient normaux et les bilans infectieux et immunologique exhaustifs sont revenus négatifs. Une rechute de lymphome a été suspectée d’où réalisation d’une TDM étagée et d’un PET-scanner au FDG qui ont mis en évidence les adénomégalies cervicales bilatérales sans autres lésions.

Le curage ganglionnaire cervical et la BOM ont permis d′éliminer le diagnostic de rechute lymphomateuse. Devant l′association polyarthrite, rash maculopapuleux évanescent, fièvre vespérale, polyadénopathie et hyperferritinémie le diagnostic de maladie de Still a été évoqué d’autant plus que la fraction glyquée de la ferritine était effondrée < 20%.

Un traitement par corticoïdes a été initié d’abord en bolus puis relais per os à 1mg/kg/j avec une réponse partielle puis adjonction du Méthotrexate à 15 mg/semaine ayant permis une bonne évolution clinique (disparition des arthrites, des poussées cutanées et de la fièvre) et biologique (normalisation des paramètres de l’inflammation, du taux de leucocytes, de la ferritinémie et de sa fraction glyquée) en 6 semaines. Après 30 mois de suivi, la patiente est toujours asymptomatique son examen clinique et son bilan biologique sont strictement normaux.

## Discussion

La maladie de Still de l’adulte est une maladie auto-inflammatoire multigénique rare et généralement de bon pronostic. Sa pathogénie se situe au carrefour nosologique entre inflammasomopathies et activation lymphohistiocytaire [1]. Son diagnostic repose sur l’association de signes cliniques (fièvre vespérale, éruption maculo-papuleuse saumonée évanescente, arthralgies ou arthrites, polyadénopathie, douleur pharyngée) et biologiques (hyperleucocytose , hyperferritinémie et fraction glycosylée de la ferritine effondrée) et l’exclusion d’une pathologie auto-immune, infectieuse ou néoplasique [2 , 3].

L’association maladie de Still et néoplasie a été décrite dans quelques observations (28 cas) rassemblées dans une revue de la literature [4]. Le plus souvent le diagnostic de maladie de Still précédait celui de la néoplasie (15 cas), et dans quelques cas les deux pathologies ont été diagnostiquées simultanément (9 cas) et seulement 4 observations de néoplasie solide ayant précédé la MSA sont rapportées, mais à notre connaissance aucune observation de maladie de Still succédant à une hémopathie et en particulier à un lymphome n’a été rapportée. Il s’agissait le plus souvent de néoplasies solides (17 cas) avec en tête le cancer du sein (6 cas); les hémopathies (11 cas) étaient surtout représentées par les lymphomes malins de phénotype B ou T (7 observations) (Tableau 1). Deux cas de syndromes myéloprolifératifs, un cas de LLC et un cas de maladie de Hodgkin ont également été rapportés [4]. La maladie de Still de l’adulte et les lymphomes sont des pathologies dont la présentation clinique et même histopathologique est très proche rendant la distinction entre ces deux entités très délicate [5].

**Tableau 1:** Maladie de Still de l’adulte etlymphomes malins: caractéristiques des 9 cas rapportés

Auteur	Age/Sexe	Lymphome associé	Chronologie (mois)	MSA ttt et réponse	Néoplasie ttt et réponse	Evolution
Trotta et al.	52/F	LMNH (grandes cellules B)	MSA précède (24)	CtC (RI)	Chimiothérapie	MSA: RI LMNH: RC
Kawasaki et al.	58/F	LMNH (cellules T)	MSA précède (4)	CtC discontinue (RI)	Chimiothérapie	MSA et LMNH: RC après Chimiothérapie
Isimbaldi et al.	19/M	LMNH (angiotrope T)	Simultanés	Ctc (échec)	0(diagnostic autopsique)	Décès/défaillance multiviscérale
Sono et al.	50/M	LMNH (grandes cellules B)	MSA précède (18)	CtC, CPM, MTX (RI)	Chimiothérapie	LMNH réfractaire
Otrock et al.	32/F	LMNH (grandes cellules)	MSA précède (10)	CtC (RC)	Chimiothérapie	MSA: controlée LMNH: rechute
Kato et al.	25/M	LMNH (nasal NK)	simultanés	CtC (RI)	Chimiothérapie	Non évaluable
Gratton et Imboden	23/F	Maladie de Hodgkin	MSA précède (1)	AINS (non évaluable)	Chimiothérapie	MSA: pas de rechute Hodgkin: RC
Voinchet et al.	38/M	LMNH splénique (zone marginale)	MSA précède (12)	AINS (échec)	Non évaluable	Non évaluable
Notre observation	26/F	LMNH( grandes cellules B)	LMNH précède(29)	CtC (RI) puis MTX (RC)	Chimiothérapie (R-CHOP: RI) puis R-ECHAP +auto-greffe avec RC	MSA: RC LMNH: RC

MSA: maladie de Still de l’adulte; ttt: traitement; LMNH : lymphome malin non hodgkinien ; CtC : corticothérapie (0,5 à 1 mg/kg/j); MTX: Méthotrexate; CPM: Cyclophosphamide; AINS: anti-inflammatoires non stéroïdiens; RC: rémission complète; RI: rémission incomplète

Le risque de survenue de lymphome est majoré chez les patients porteurs de maladies auto-immunes [6, 7]. La prévalence de survenue d’un lymphome parait plus importante au cours du Syndrome de Sjörgen (risque x15), du Lupus érythémateux systémique (risque x4), de la polyarthrite rhumatoïde ( risque x2) et de la maladie cœliaque [6, 7]. Par ailleurs, certains lymphomes non Hodgkiniens peuvent s’accompagner de manifestations dysimmunitaires notamment musculosquelettiques, cutanées, hématologiques ou même neurologiques suggérant que lymphoprolifération et auto-immunité constituaient deux facettes d’un même processus [8].

La maladie de Still associée aux néoplasies répond généralement aux critères diagnostics de Yamagushi [2], cependant quelques atypies doivent alarmer sur la possibilité d’une néoplasie sous-jacente notamment: l’âge de survenue inhabituel, installation rapide des symptômes, mauvaise réponse à la corticothérapie ou aux immunosuppresseurs ou certaines anomalies biologiques(cytopénies, hypergammaglobulinémie importante) [4].

Notre observation présente la particularité du diagnostic de maladie de Still 18 mois après traitement d’un lymphome à grandes cellules B par chimiothérapie et auto-greffe avec rémission complète. La symptomatologie répondait aux critères de Yamagushi [2] et à ceux de Fautrel [3]. Une rechute lymphomateuse a été évoquée en premier mais a pu être écartée de même qu’une pathologie infectieuse ou auto-immune.

Nous avons alors émis les hypothèses suivantes: la maladie de Still existait-elle avant même le lymphome, vu la présentation clinique initiale, mais refroidie par la chimiothérapie, ou a-t-elle été favorisée par le terrain et /ou les traitements reçus ou même par une infection greffée sur ce terrain immunodéprimé ou alors constituerait-elle un syndrome paranéoplasique auquel cas elle serait annonciatrice d’une rechute. Après un recul de 30 mois la patiente est toujours asymptomatique sous faibles doses de corticoïdes et Méthotrexate 15mg/semaine ce qui conforte plutôt l’hypothèse d’une simple association des deux pathologies sur un terrain certes particulier.

## Conclusion

Le diagnostic de maladie de Still reste un diagnostic d’exclusion mais qui doit être considéré devant un tableau clinico-biologique évocateur et après une enquête étiologique rigoureuse permettant d’écarter une origine néoplasique, infectieuse ou auto-immune. Les liens immunopathologiques entre maladie de Still et lymphome ne sont pas encore élucidés surtout que les cas rapportés sont très rares. Une vigilance est cependant de mise devant toute atypie du tableau clinique ou de résistance au traitement lors d’une maladie de Still.

## Conflits d’intérêts

Les auteurs ne déclarent aucun conflit d'intérêts.
